# Diagnosis of Colorectal Cancer and Adenomatous Polyps of the Colon Based on the Level of MicroRNA Expression in the Mucous Membrane (Pilot Clinical Study)

**DOI:** 10.17691/stm2024.16.5.05

**Published:** 2024-10-30

**Authors:** M.V. Bagryantsev, A.A. Yanyshev, M.G. Ryabkov, A.I. Abelevich, I.L. Dezortsev, A.V. Bazayev

**Affiliations:** MD, PhD, Surgeon; Nizhny Novgorod Regional Clinical Hospital named after N.A. Semashko, 190 Rodionova St., Nizhny Novgorod, 603126, Russia; MD, PhD, Tutor, Department of General, Operative Surgery and Topographic Anatomy; Privolzhsky Research Medical University, 10/1 Minin and Pozharsky Square, Nizhny Novgorod, 603005, Russia; Surgeon; Nizhny Novgorod Regional Clinical Hospital named after N.A. Semashko, 190 Rodionova St., Nizhny Novgorod, 603126, Russia; MD, DSc, Associate Professor, Chief Researcher, Laboratory of Optical Coherent Tomography, Research Institute of Experimental Oncology and Biomedical Technologies; Privolzhsky Research Medical University, 10/1 Minin and Pozharsky Square, Nizhny Novgorod, 603005, Russia; MD, DSc, Professor, Department of General, Operative Surgery and Topographic Anatomy; Privolzhsky Research Medical University, 10/1 Minin and Pozharsky Square, Nizhny Novgorod, 603005, Russia; MD, PhD, Head of the Coloproctology Department; Nizhny Novgorod Regional Clinical Hospital named after N.A. Semashko, 190 Rodionova St., Nizhny Novgorod, 603126, Russia; MD, DSc, Head of the Department of General, Operative Surgery and Topographic Anatomy; Privolzhsky Research Medical University, 10/1 Minin and Pozharsky Square, Nizhny Novgorod, 603005, Russia

**Keywords:** colorectal cancer, adenomatous polyps, colon mucous membrane, microRNA expression

## Abstract

The gold standard for colorectal cancer (CRC) diagnosis, colonoscopy with biopsy, is an invasive technique and has some limitations, while the known non-invasive methods do not possess sufficient sensitivity and specificity. The application of microRNA as a diagnostic and prognostic CRC biomarker may compensate for the colonoscopy limitations. However, there are no data in the literature on the existence of real test systems based on the evaluation of microRNA expression. Our pilot study is the first step to creating a test system for CRC diagnosis based on the analysis of microRNA expression in the tissue of the intact colon.

**The aim of the study** is to assess the prospects of using the level of microRNA expression as a supplemental method for diagnosing colorectal cancer and adenomatous polyps.

**Materials and Methods:**

Patients participating in the study were divided into three groups: group 1 included patients with CRC (n=5), group 2 — patients with polyps in the colon (n=4), and patients without oncological pathology treated for hemorrhoidal disease without exacerbation (n=5) composed group 3.

Tissue samples of the intact gut were taken from all patients. In groups 1 and 2, biopsy was performed in the process of right-sided laparoscopic resection of the colon with tumor. Samples of the mucous membrane from the distal part of the rectum in patients of group 3 were also collected intraoperatively; they were operated on using Milligan-Morgan technique. In groups 1 and 2, CRC and polyp samples, respectively, were taken for the analysis additionally to the intact gut area.

The test panel included the following microRNAs: hsa-miR-10b-5p, hsa-miR-20a-5p, hsa-miR-141-3p, hsa-miR-181b-5p. The levels of the reference genes were analyzed with the help of real-time polymerase chain reaction using intercalating SYBR Green stain.

**Results:**

Expression of hsa-miR-141-3p in the mucous membrane of the colon in patients of groups 1 and 2 (with CRC and polyps, respectively) was statistically significantly higher than in patients without bowel tumors. At the same time, the expression level of hsa-miR-10b-5p was statistically significantly lower in the tumor tissue (cancer or polyps) in comparison with patients of group 3.

Lower values of expression in all tested microRNAs have been detected in the CRC tissue relative to the intact mucosa of the same patients. A similar tendency was also observed in patients with adenomatous polyps.

**Conclusion:**

The results of the study have shown that of four microRNAs, included into the test panel, hsa-miR-141-3p and hsa-miR-10b-5p were found to have the diagnostic value for identifying tumor colorectal lesions. Thus, our data will assume that supplementing the endoscopic tests of the large intestine by the epigenetic analysis of the mucous membrane is a promising approach to cancer screening procedures.

## Introduction

According to the WHO prognosis, the number of first-detected colorectal cancers (CRC) will grow to 3.2 million new cases per year by 2040, being a 63% increase from 2023 [1]. Early diagnosis is a priority direction for CRC-related lethality reduction, while the development and implementation of diagnostic systems with high sensitivity and specificity for timely verification of this diagnosis is really a burning task.

The list of the main non-invasive methods of early CRC diagnosis is included into clinical recommendations in many countries and contains fecal occult blood test (FOBT with the sensitivity of 50–80% and specificity 85–97%) [2], fecal immunochemical test (FIT with 61–91% sensitivity and 91–98% specificity) [3], and fecal DNA test (mt-sDNA, Cologuard® with 92% sensitivity and 94% specificity) [4]. These tests aim to detect blood or tumor cell fragments in the intestinal contents. Their key drawbacks are relatively low indicators of CRC detectability especially in the right-sided localization of the tumor, which causes the necessity of frequent screenings [5].

Sygmoidoscopy, colonoscopy, CT colonography, and capsule endoscopy are the main invasive methods of direct imaging used as CRC screening techniques [6]. Colonoscopy and sigmoidoscopy are highly specific and sensitive methods and for this reason are recognized as the gold standard in CRC diagnosis [7]. However, colonoscopy requires a thorough and long-term preparation of the large intestine for the examination, sedation of patients, whereas diagnostic sensitivity and specifcity vary depending on the technical characteristics of the equipment and endoscopists’ expertise level. An essential part of patients refuses to undergo colonoscopy for fear; in some cases, which are large in number, the procedure is contraindicated due to adhesions in the abdominal cavity [8, 9]. These factors restrain mass and widespread use of colonoscopy as a non-alternative, comprehensive method of the large intestine tumor screening. As the result, the frequency of the existing but not diagnosed (interval) CRC reaches 4–9% [10, 11]. For these reasons, the need for developing and implementing auxiliary screening methods for early diagnosis of intestinal tumors does not give rise to doubts.

With the development of omics technologies there appeared a new perspective in improving the efficiency of CRC screening. Presently, changes in the microRNA expression activity in the gut tissues in CRC are being rather intensively investigated [12–14], however, we did not manage to find any literature data on the test systems developed and introduced into clinical practice, which could verify CRC based on microRNA expression. We assume that the combination of colonoscopy with determination of microRNA expression level in the colonic mucosa will allow the development of more informative procedure for early CRC diagnosis. This, in turn, will help reduce significantly the number of interval CRC. In order to test this hypothesis, it is necessary to answer the question whether there are differences in microRNA expression in the colonic mucosa in patients with CRC and polyps in the colon and patients with oncological diseases of the large intestine. Our pilot clinical study is just devoted to the solution of this question.

**The aim of the study** is to assess the prospects of using the level of microRNA expression as a supplemental method of diagnosing colorectal cancer and adenomatous polyps.

## Materials and Methods

Samples of colonic tissues and biological fluid have been investigated. The material was taken from 14 patients: 8 men (57%) and 6 women (43%); median age — 67 [61; 74] years. Five patients were treated for CRC (group 1); 4 patients were found to have benign polyps in the colon (group 2); 5 patients did not have oncological pathology in the colon, they were treated for hemorrhoidal disease without exacerbation (group 3, control).

21 samples of colonic tissue have been examined: 5 samples of the intact tissue and 4 samples of the tumorous tissue in group 1; 4 samples of the intact tissue and 3 samples of the polyp tissue in group 2; 5 samples of the intact colonic mucosa in group 3. The expression levels of the tested microRNA in the colonic mucosa were compared between the groups. Additionally, microRNA expression in the tissues of CRC (group 1) and adenomatous polyp (group 2) was compared with that of the tissue of the intact mucosa obtained from the patients of group 3.

Patients with the complicated course of the diseases such as intestinal obstruction, bleeding, tumor perforation, and other possible complications, were excluded from the study.

Written informed consent was obtained from all patients for participation in the study including biopsy of colonic mucous membrane. The study was carried out in compliance with the Declaration of Helsinki and approved by the Ethics Committee of Privolzhsky Research Medical University (Protocol Code No.9, the date of approval: June 10, 2022).

**Figure 1.**
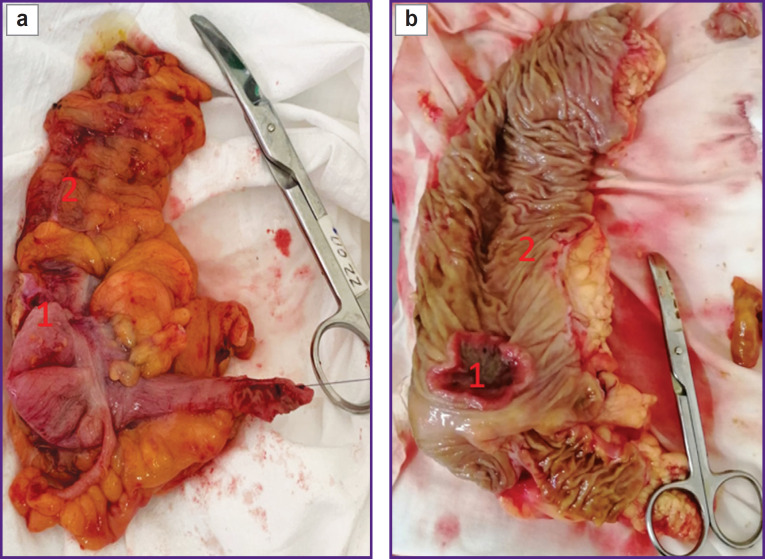
Preparation — the right side of the colon with a tumor and indication of the sample collection site: (a) integral preparation; (b) preparation after gut dissection; 1 — tumor of the ascending colon; 2 — unchanged region of the ascending colon, sites of sample collection

Samples of the gut intact tissue were collected from all patients. Biopsy in patients of groups 1 and 2 was performed in the process of right-sided laparoscopic resection of the colon with tumor. During laparoscopic right-sided hemicolectomy and removal of the preparation from the abdominal cavity (Figure 1), a colon lumen was opened through a mid-median minilaparotomy and a 0.5×0.5 cm sample of the mucous membrane, localized at a distance not less than 10 cm from the tumor, was taken under visual control. The size of the tissue area corresponded to the standard dimensions of the bioptate obtained with the help of biopsy forceps during colonoscopy.

Persons included into group 3 were treated for grade II–III chronic hemorrhoid and operated on using Milligan– Morgan technique. Samples of the mucous membrane from the distal part of the rectum 0.5×0.5 cm in size were collected also intraoperatively. In groups 1 and 2, samples of CRC and polyps were taken for analysis additionally to the fragment of the intact gut.

Apart from routine laboratory and instrumental methods of examination (general blood test, general analysis of urine, biochemical blood analysis, coagulogram, electrocardiogram, etc.), patients underwent colonoscopy with histological verification of the diagnosis, gastroscopy, abdominal and thoracic intravenous contrast-enhanced CT, and pelvic MRI; biomarkers for CRC (carcinoembryonic antigen and CA 19-9) were also identified.

The list of tested microRNAs was determined based on the literature data [15]: hsa-miR-10b-5p, hsa-miR-20a-5p, hsa-miR-141-3p, hsa-miR-181b-5p. MicroRNA hsa-miR-10b-5p regulates transcripts CDKN1A, CDKN2A, BCL2L11, PTCH1, TP53; microRNA hsa-miR-20a-5p — TP53INP1, CDKN1A, E2F1, PPP2R2A, TGFBR2, BCL2; microRNA hsa-miR-141-3p — MAP4K4, PTEN, WDR37, CDC25A, KLF5; microRNA hsa-miR-181b-5p — MCL1, BCL2, TCL1a, XIAP.

MicroRNAs were isolated from the clinical samples using HiPure Universal miRNA Kit (Magen Biotechnology Co., Ltd, China). The level of microRNA expression was analyzed with the help of ALMIR kits (Aligmed Techno, Belarus) according to the manufacturer’s recommendations using the CFX96 Touch real-time PCR detection system (Bio-Rad, USA). MicroRNA expression for the tissue samples was normalized relative to the set of the reference genes (*EIF2B1*, *IPO8*, *ABL1*) determined by the geNorm algorithm [16, 17]. The levels of the mRNA reference genes were analyzed by means of real-time PCR using intercalating SYBR Green stain.

### Statistical analysis

Data was statistically processed using the IBM SPSS Statistics 20 software package. The normality of quantitative variable distribution was checked by the Kolmagorov–Smirnov test. All examined indicators were not normally distributed, therefore, to assess statistical significance of differences when comparing the groups by quantitative features, nonparametric methods were applied. The Kruskal– Wallis test was used to compare the values in the groups. Sampling parameters given below are designated as follows: Me (median), Q1 (upper quartile), Q3 (lower quartile), min and max (minimal and maximal value of the variable), n (a volume of the analyzed subgroup), p (the value of statistical significance of differences). The critical value of the significance level was considered 5% (p≤0.05). When multiple comparisons were used, the level of difference significance is indicated as a corrected value (p_adjusted_=p·m, where p is the value obtained from the results of comparison, m — is the number of comparisons).

## Results

### Expression of microRNA in the intact colonic mucosa in patients of all groups

**Figure 2.**
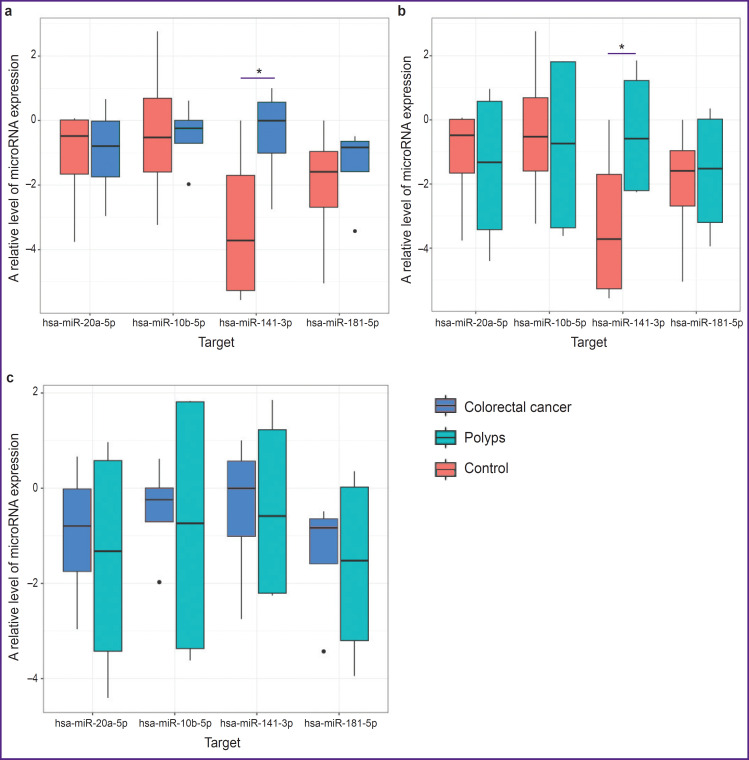
A relative level of microRNA expression in the intact samples of the gut tissue: (a) samples of groups 1 and 3; (b) samples of groups 2 and 3; (c) samples of groups 1 and 2; * statistically significant differences between the groups, p<0.05

Using multiple comparisons of groups 1, 2, and 3 (patients with CRC, polyps, and hemorrhoidal disease), statistically significant differences of the hsa-miR-141-3p expression level (Kruskal–Wallis test, p=0.032) have been found in the tissue of the intact gut. Pairwise comparison has detected that hsa-miR-141-3p expression in groups 1 and 2 is statistically significantly higher than in group 3 (Figure 2 (a), (b)) (p=0.032). Although no statistically significant differences in this indicator were found when group 1 and 2 were compared (bioptates of patients with CRC and polyps) (Figure 2 (c)).

Statistically significant differences between the groups in the expression level of hsa-miR-10b-5p, hsa-miR-20a-5p, hsa-miR-181b-5p have not been identified in the samples of the intact tissue of the gut (see Figure 2).

### MicroRNA expression in the tumor tissue of groups 1 and 2 (CRC and polyps) and the tissue of the normal mucous membrane in group 3

**Figure 3.**
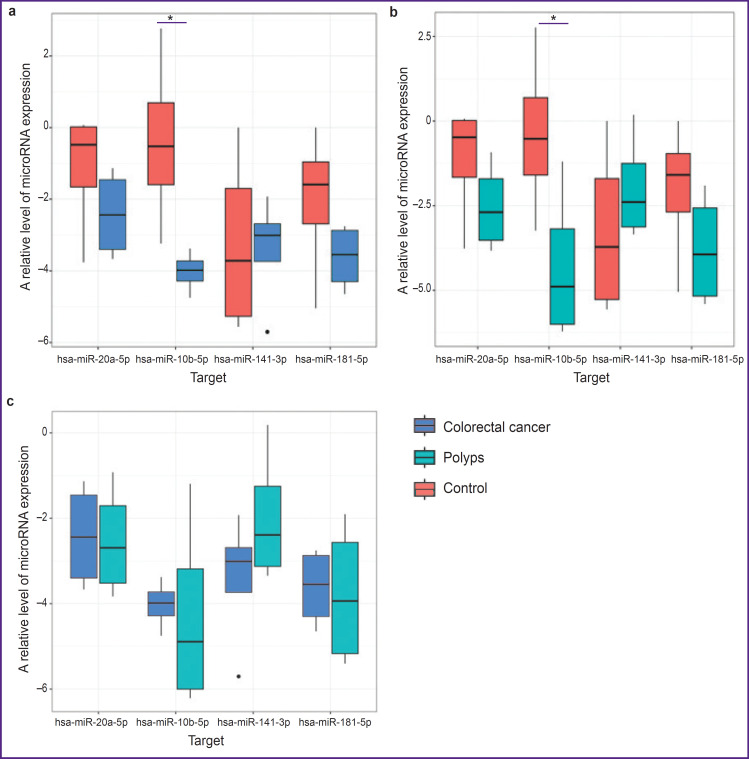
A relative level of microRNA expression in the samples of colorectal cancer and polyp relative to the normal tissue: (a) samples of groups 1 and 3; (b) samples of groups 2 and 3; (c) samples of groups 1 and 2; * statistically significant differences between the groups, p<0.05

When comparing the indicators of microRNA expression, there was observed a unidirectional tendency: the expression levels of hsa-miR-10b-5p, hsa-miR-20a-5p, hsa-miR-141-3p, hsa-miR-181b-5p in the tissues of patients in group 3 were higher than in the CRC and polyp tissues (Figure 3 (a), (b)). Statistically significant differences were found only in the expression level of hsa-miR-10b-5p when comparing groups 1 and 3 (p_adjusted_=0.018; Figure 3 (a)) and groups 2 and 3 (p_adjusted_=0.011; Figure 3 (b)). At the same time, when comparing the expression level in the malignant tumor and in the polyp, statistically significant differences were not detected (Figure 3 (c)).

### MicroRNA expression in the tumor and intact tissue in groups 1 and 2

**Figure 4.**
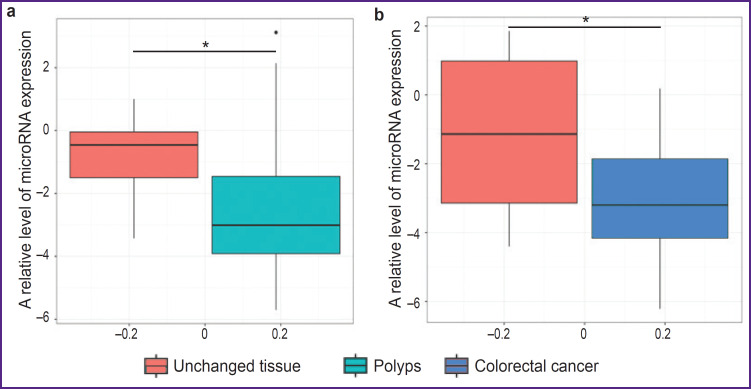
A box plot of expression levels of all microRNA variants: (a) intact tissue and polyps; (b) intact tissue and colorectal cancer; * statistically significant differences between the groups, p<0.05

Lower values of expression of the four tested microRNAs were obtained in the CRC tissue (Figure 4) as compared to the intact mucosa of the same patients (p_adjusted_=0.033). A similar tendency was also observed in patients with adenomatous polyps (p_adjusted_=0.045).

Thus, a general effect of microRNA expression reduction has been observed in the tumor tissue (CRC and polyps) relative to the intact mucous membrane. Therefore, the above decrease of has-miR-10b-5p in the tumor tissue may be the result of this effect.

## Discussion

Only two epigenetic commercial diagnostic kits, used in practice to analyze gene expression with the help of methylated DNA, SEPT9 and VIM, have been registered in the world by 2024 [18, 19]. It should be noted that one of them, the test for detecting methylated SEPT9 (Epi procolon®) gene, has a relatively low sensitivity (48%) at 92% specifcity. The sensitivity of this method in the diagnosis of suspicious adenomas is even lower [20]. Thus, investigations in the field of designing diagnostic tests based on the analysis of microRNA expression for CRC diagnosis are presently of vital importance [15, 21–24].

An increased level of hsa-miR-141-3p expression may designate a favorable prognosis of CRC course, since the decrease of the level facilitates the development of a more aggressive tumor, its fast growth, and metastasizing. The level of hsa-miR-10b-5p expression is associated with the activity and proliferation of tumor cells. According to the literature data, expression correlates with the stage of tumor development [25, 26], which may serve as a validation of a high risk for its malignant transformation. It is noteworthy that the intact tissue obtained from the patients with polyps has a high heterogeneity and clear clustering into the samples with an increased and decreased expression relative to the normal tissue, which may speak of a risk for malignization of an adenomatous polyp.

## Conclusion

Of four microRNAs (hsa-miR-10b-5p, hsa-miR-20a-5p, hsa-miR-141-3p, hsa-miR-181b-5p), included into the test panel, hsa-miR-141-3p and hsa-miR-10b-5p are of a diagnostic value for revealing tumor lesions of the large intestine. Expression of hsa-miR-141-3p in the large gut mucous membrane in patients with colorectal cancer and patients with polyps is statistically significantly higher than in patients without oncological bowel diseases. At the same time, the comparison of the hsa-miR-10b-5p expression level in the tumor tissue (cancer and polyp) with the tissue of the gut mucous membrane in patients without bowel tumors has shown an opposite result: this indicator is reliably lower in the tissue of colorectal cancer and adenomatous polyp. All tested microRNAs in the intact tissue are characterized by significantly higher level of expression in comparison with the tumor tissue. Thus, the data obtained in our pilot study confirm the idea that endoscopic examination of the large bowel supplemented with an epigenetic analysis of the mucous membrane may be a promising approach to cancer screening tests.
